# Ideal Time for the Placement of Intravenous Catheter in Children Following the Induction of Anaesthesia With Sevoflurane in Nitrous Oxide and Oxygen Without Any Premedication

**DOI:** 10.7759/cureus.97994

**Published:** 2025-11-28

**Authors:** Anushta Paul, Arindam Phukan, Arbind K Ray, Diganta Saikia, Karuna K Das

**Affiliations:** 1 Department of Anaesthesiology, Assam Medical College and Hospital, Dibrugarh, IND

**Keywords:** cannulation, children, ideal time, nitrous oxide, paediatric anesthesia, sevoflurane

## Abstract

Background

Children scheduled for surgical procedures who don’t have a pre-placed or functional Intravenous Catheter (IVC) often undergo inhalational induction prior to attempting Intravenous Catheter Placement (IVCP). An optimum depth of anaesthesia is necessary to prevent adverse consequences of painful stimuli. Here, it becomes important to correlate the optimal time following induction with that of optimal depth of anaesthesia to carry out IVCP in children. We endeavoured to determine the ideal time for IVCP in un-premedicatedchildren following inhalational induction of anaesthesia.

Methods

Ours was a prospective, up-down sequential allocation, observer-blinded study conducted on 106 un-premedicated children, aged 1 to 10 years. The IVCP time was set at 3 minutes after the loss of eyelash reflex for the first child following induction with sevoflurane and nitrous oxide in oxygen using a single-step approach, and the time for cannulation was determined by using a step-up-down size of 15 seconds initially and 5 seconds later on. The time of IVCP was considered ideal if there was no patient movement or cough while attempting.

Results

The mean ideal time for cannulation after loss of eyelash reflex following inhalational induction was 56.75 seconds (53.58 - 59.89 seconds) in 50% patients and 73.77 seconds (69.67 - 77.88 seconds) in 95% patients with 95% confidence limits.

Conclusion

A waiting period of 1 min 22 seconds (82 seconds) after the loss of eyelash reflex in un-premedicated children facilitates smooth IVCP following sevoflurane induction in nitrous oxide and oxygen by the single-step approach.

## Introduction

Intravenous catheter placement (IVCP) is mandatory for any surgical procedure under anaesthesia in children of any age. IVCP is painful and may produce long-term psychological and physiological consequences in children and newborns. These children also frequently develop hyperalgesia [1]. Furthermore, the majority of the older children resist attempts to IVCP while awake, necessitating multiple attempts or subjecting them to trauma. To mitigate these adverse consequences, inhalational induction with volatile anesthetic agents is carried out in children lacking a pre-existing or functional IVC, before any surgical procedure under anaesthesia. Sevoflurane is a widely approved agent of choice for mask induction in the paediatric population owing to its non-irritant nature, hemodynamic stability, and lower pungency [2]. Nitrous oxide is co-administered to shorten the second stage of anaesthesia [3].

Early attempt at cannulation during inhalational induction may lead to undesired movements, cough, breath holding, and even laryngospasm, which can become fatal if not promptly managed [4], whereas unnecessary delay in IVCP may impede safe management of apnoea and can lead to hypoxia and bradycardia [5]. Thus, it is crucial to assess the optimum depth of anaesthesia following exposure to volatile agents in these children. Since loss of consciousness and observable physical parameters do not correlate with depth of anaesthesia with modern anaesthetics, it becomes important for an anesthesiologist to correlate the optimal timing of exposure with that of an optimal depth of anaesthesia to carry out interventional procedures in children. A limited amount of literature exists on the ideal timing for placement of intravenous cannulation in the paediatric population following inhalational anaesthesia.

In this study, our primary objective was to determine the ideal time for IVCP in un-premedicated children following inhalational induction with sevoflurane and nitrous oxide in oxygen using the single-step approach. While our secondary objective was to determine the duration required to achieve loss of eyelash reflex during inhalational induction, the mask acceptance score, and the detection of movement at the time of IVCP.

## Materials and methods

**Figure 1:**
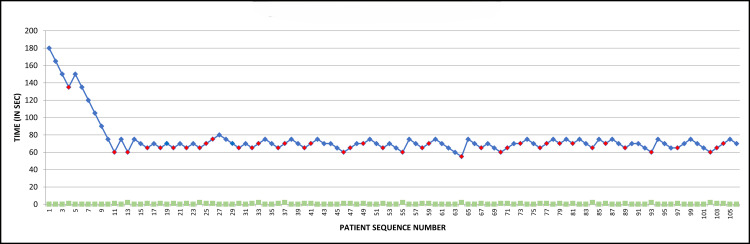
The sequential results of Dixon’s up and down method showing loss of eyelash reflex to cannulation time. This line diagram depicts the sequential results plotted with respect to the time of inhalational induction for successful intravenous catheter placement (IVCP) following loss of eyelash reflex in 106 children. The data of each patient is represented by a square block. A blue square block represents no movement, while a red square block represents movement at the time of intravenous cannulation. The green square blocks in the X-axis represent the patient sequence number from 1 to 106.

After obtaining approval from the Institutional Ethics Committee of Assam Medical College (registration number: ECR/2003/Inst/AS/2024, approval number: 2025AMC/EC/1236, dated February 24, 2025) we conducted an observer blinded, prospective, up-and-down sequentially allocated study (Fig. 1), in un-premedicated children aged 1 to 10 years, undergoing elective paediatric surgery from March 2025 to May 2025 in a tertiary healthcare centre in Northeast India. Written and informed consent was acquired from the parents, and the study continued until the predetermined sample size of 106 was achieved.

Study subjects were selected on the basis of the following inclusion and exclusion criteria.

Inclusion criteria

Patients without a pre-existing or functional intravenous cannulation, belonging to American Society of Anaesthesiologists (ASA) grade I and II, undergoing elective paediatric surgery were included in the study.

Exclusion criteria

Those with congenital airway anomalies, anticipated difficult intubation, history of respiratory infections in the last four weeks, full stomach, patients with a history of intraoperative laryngospasm or bronchospasm, patients with hypovolemia, and those undergoing treatment with sedatives or anticonvulsant agents, or belonging to ASA grade III and higher, were excluded from the study.

The patients were shifted to the operation table, standard monitors were applied, and all vital parameters such as peripheral oxygen saturation (Spo2), heart rate (HR), ECG, and end-tidal carbon dioxide (EtCO2) were monitored continuously. The Jackson Rees (JR) circuit was then flushed and refilled with sevoflurane (8%) in nitrous oxide and oxygen (50%:50%) for 30 seconds with a fresh gas flow (FGF) of 6 l/min [4]. The face mask was gently introduced in front of the patient, enabling them to naturally breathe in the anesthetic gaseous mixture spontaneously from their immediate environment. As the child started getting drowsy, the timer was set. The face mask was then appropriately positioned, and the Mask Acceptance Score (MAS) was evaluated. At the loss of eyelash reflex, the time was noted.

The cannulation trial for the first child was initiated at 3 minutes following the disappearance of eyelash reflex. It was then stepped up and down by 15 seconds initially and 5 seconds later, depending on negative or positive response, respectively, in the preceding child. Spontaneous respiration with the preset gaseous mixture was permitted till successful IVCP was performed by an experienced paediatric anesthesiologist. Inhalation of nitrous oxide was thereafter discontinued, and the concentration of sevoflurane was adjusted in accordance with the patient’s condition, and ventilation was gently aided as required. Movement of the patient at the time of cannulation was observed minutely and categorized by an independent observer. The time required to cannulate and the attempts of cannulation were also noted. If there was no movement, coughing, or laryngospasm, then the timing of cannulation was considered ideal.

Sample size calculation

Despite an extensive PubMed search, no published literature was found regarding the prevalence of children undergoing intravenous catheterization under inhalational anaesthesia in the paediatric population of 1 to 10 years of age. Thus, assuming 50% prevalence (P=0.5), 10% absolute error (L = 0.1), 95% confidence interval, 10% dropouts, using the formula $\frac{Z^{2}\, p\, (1 - p)\, (1.1)}{L^{2}}$, the minimum effective sample size was 106.

Statistical methods

The Probit test, a specialized logistic regression for binomial responses, was employed to determine the optimal time for IVCP in 50% and 95% of patients with 95% confidence limits (CL) of the mean from an up-and-down sequence [6]. IBM SPSS Statistics for Windows, Version 20.0 (IBM Corp., Armonk, USA) was employed to evaluate the data. The mean standard deviation (SD) was used to express the demographic and clinical data. The hemodynamic alterations between the preoperative period and inhalational induction were compared using the unpaired t-test.

Outcome measures

Primary Outcome Measure

The primary outcome measure - the ideal time for intravenous catheter placement (IVCP) - was determined by employing Dixon's up-and-down sequential method, commencing at 3 minutes for the first child [7].

Secondary Outcome Measures

The following were the secondary outcome measures: (1) the duration required to achieve loss of eyelash reflex during inhalational induction; (2) the mask acceptance score in children was recorded using a four-point scale, in which 1 was assigned to children who were combative and crying, 2 for children with moderate fear of mask, 3 for children who co-operated with reassurance, and 4 was assigned to children who were calm, co-operative or asleep [8]; and (3) the detection of movement at the moment of cannulation was accomplished using a four-point scale in which 0 denotes no movement, 1 for those exhibiting slight extremity tension, 2 for extremity withdrawal and 3 in case of generalized movement [9].

## Results

The mean age of the children in our study population was 5.35 ± 2.95 years, with a mean body weight of 15.06 ± 5.78 kg. Out of 106 children, 76 were male, and 30 were female. The change in heart rate of the study population during inhalational induction as compared to the pre-operative period by unpaired t-test turned out to be insignificant in statistical terms (p=0.119). Similarly, no significant variation was observed in oxygen saturation (p=0.346).

In our study, the average time required to achieve loss of eyelash reflex was 39.20 ± 5.50 seconds. The ideal time for IVCP following loss of eyelash reflex using Dixon’s method in 50% of our patients, when induced with sevoflurane and nitrous oxide in oxygen, was 56.75 seconds (53.58 - 59.89 seconds), while it was 73.77 seconds (69.67 - 77.88 seconds) in 95% of patients with a 95% confidence interval. The mean attempt of cannulation was 1.11 ± 0.32, and 17.96 ± 6.03 seconds was the mean time required to perform IVCP.

The cannulation trial for the first child was initiated at 3 minutes (180 seconds) following the loss of eyelash reflex. It was then stepped up and down by 15 seconds initially and 5 seconds later, depending on negative or positive response, respectively, in the preceding child. The movement at the time of cannulation was noted, and the graphical representation of the data thus obtained has been put together in Fig. 1.

The mask acceptance score of the children is described using a four-point scale (Table 1). While most of the children were cooperative with reassurance, a significant percentage had moderate fear or were combative; however, some were calm and cooperative as well.

**Table 1:** Mask acceptance score of the children The table shows the mask acceptance score: 16.04% were combative to the mask, 27.36% showed moderate fear of the mask, 48.11% were cooperative with reassurance, while 8.49% were calm and cooperative.

Mask Acceptance Score (MAS)	Physical parameter	Number of children	Percentage of children
1	Combative, crying	17	16.04%
2	Moderate fear of the mask, not easily calmed	29	27.36%
3	Co-operative with reassurance	51	48.11%
4	Calm, cooperative, asleep	9	8.49%

The movement at the time of cannulation was tabulated (Table 2). Most of the children did not move at the time of IVCP, while slight extremity tension and withdrawal were observed in those children, whose depth of anaesthesia was not adequate, presumably due to less-than-optimal exposure to volatile anaesthetic.

**Table 2:** Movement at the time of cannulation The table describes the movement at the time of cannulation: 60.38% children had no movement at the time of intravenous catheter placement (IVCP), 31.13% had slight extremity tension, while only 8.49% had extremity withdrawal.

Score	Movement at the time of cannulation	Number of children	Percentage of children
0	No movement	64	60.38%
1	Slight extremity tension	33	31.13%
2	Extremity withdrawal	9	8.49%
3	Generalised movement	0	0%

## Discussion

Our study intended to determine the duration necessary to obtain a sufficient depth of anaesthesia for successful placement of an intravenous catheter in un-premedicated children following inhalational induction with sevoflurane in nitrous oxide and oxygen in 106 un-premedicated children aged 1 to 10 years undergoing elective paediatric surgical intervention.

We observed that 50% of our patients allowed smooth placement of intravenous catheter after 56.75 seconds (53.58 - 59.89 seconds) following loss of eyelash reflex, while 95% of patients allowed the same after 73.77 seconds (69.67 - 77.88 seconds).

Hasan et al. conducted a study with a similar research methodology to ours and observed that 53.02 seconds in 50% children and 87.21 seconds in 95% children were adequate for IVCP and consequently proposed that a 105-second waiting time should precede the attempt at cannulation [4].

In our study, the time taken to achieve loss of eyelash reflex was 39.20 ± 5.50 seconds, which is close to 46.85 ± 8.76 seconds obtained by Hasan et al. [4]. Dubois et al. worked on induction techniques with incremental sevoflurane in children and observed that the addition of nitrous oxide resulted in a more rapid loss of eyelash reflex at 46 ± 9 seconds, in comparison to 61 ± 12 seconds without it [10].

The Mask Acceptance Score (MAS) was 1 in 16.04%, 2 in 27.36%, 3 in 48.11%, and 4 in 8.49% in our study population. Whereas, Hasan et al. observed a MAS of 1 in 41.2%, 2 in 17.6%, and 4 in 41.2% of patients [4]. In comparison to his study, we obtained shorter, smoother, and more effective cannulation times, along with better mask acceptance scores. This may be due to differences in the ethnicity of our study populations. The anxiety and fear varied with the age of the children and past traumatic experiences.

While observing patient movement on attempt to IVCP, 60.38% children in our study population had a score of 0 (no movement) at cannulation, 31.13% had a score of 1 (slight extremity tension), and only 8.49% of children had a score of 2 (extremity withdrawal). Whereas, Hasan et al. obtained a score of 0 (no movement) in 70.5% of the patients [4]. Ben Ali et al. in their study observed greater patient movement when IVCP was attempted at 60 seconds rather than at 90 seconds or 120 seconds following the disappearance of eyelid reflex [11].

Joshi et al. also assessed the optimal time for intravenous cannulation following induction with sevoflurane alone in oxygen. They reported an optimal time of 212 seconds, which is twice ours [12]. This may be due to the non-inclusion of nitrous oxide in their study. Nitrous oxide is known to reduce excitatory movements and cut short the second stage of anaesthesia [12].

Schwartz et al. compared the impact of IVCP at 30 seconds and 120 seconds following loss of eyelash reflex and concluded that the older and heavier children had greater susceptibility to laryngospasm [9]. We encountered continuous cough and irritation in a four-year-old child, weighing 15 kgs, with no prior history of reactive airway disease. The inhalational induction in this case was immediately discontinued. Apart from this, we did not encounter any further instances of adverse respiratory events over the duration of our study.

Premedication with midazolam is safe and effective at alleviating preoperative anxiety in children; however, in a research conducted by McCluskey and Meakin, the time to recovery from anaesthesia was significantly prolonged in children in the midazolam-premedicated group than in the un-premedicated group (28.2 vs 21.9 minutes) (p<0.05) [13]. The children in our study were predominantly uncooperative, maybe because of the absence of premedication. It is assumed that they were able to generate a greater minute volume while crying, which resulted in a more rapid induction time.

The limitations of the current study include its single-center design, small sample size, and children of the age range of only 1 to 10 years, exhibiting considerable inter-individual anatomical and physiological variations. Observer bias and cannulation bias were possible influencers during the execution of the study.

## Conclusions

Painless IVCP is a quintessential part of paediatric anaesthesia. The need for a safe, convenient, and feasible plan of anaesthesia to minimize pain and attenuate adverse intraprocedural events is inevitable. Our prospective observational study adopted an observer-blinded, sequential up-and-down allocation approach in 106 un-premedicated children planned for IVCP following gas induction, belonging to the age group of 1 to 10 years in a tertiary healthcare centre in Northeast India. The mean ideal time for cannulation after loss of eyelash reflex following inhalational induction was 56.75 seconds in 50% patients and 73.77 seconds in 95% patients. We thereby conclude that a duration of 1 minute 22 seconds (82 seconds) after the loss of eyelash reflex following a single-step induction of anaesthesia with sevoflurane (8%) and nitrous oxide: oxygen = 50: 50 allowed smooth IVCP in un-premedicated children.
